# Minipatellar Tunnels for Transosseous Fixation of Medial Patellofemoral Ligament Graft Using High-strength Suture

**DOI:** 10.1016/j.eats.2024.103100

**Published:** 2024-07-08

**Authors:** Yizhong Peng, Hong Wang, Wenbo Yang, Wei Yu, Chunqing Meng, Wei Huang

**Affiliations:** Department of Orthopaedics, Union Hospital, Tongji Medical College, Huazhong University of Science and Technology, Wuhan, China

## Abstract

Patellar dislocation is a common knee injury, with concomitant pathoanatomical risk factors that synergistically interact and predispose to patellofemoral instability. Medial patellofemoral ligament (MPFL) reconstruction has demonstrated significant potential in the re-establishment of MPFL anatomic and biological function, with low patellar redislocation rates. Although many techniques for MPFL reconstruction have been developed, challenges such as patella fractures and high costs persist. Herein, to further reduce bone defects and ensure the reliability of fixation, we developed a microbone tracts technique for MPFL reconstruction on the patella side using high-strength sutures. This technique passes high-strength sutures through the microtransosseous tunnels to fix the tendon graft on the patella side, aiming to achieve minimized patella damage with no additional implants for graft fixation, while resuturing the fascia on the surface of the patella with the suture ends further strengthens the graft fixation. This technique provides an economic and reliable solution for graft fixation on the patella with minimal bone disruption.

Patella dislocation accounts for 2% to 3% of all knee injuries.^1^^,^^2^ The incidence of patellar dislocation is relatively high, approximately 42 in 100,000, and is more commonly observed in girls between the ages of 10 and 17 years.^2^^,^^3^ Recurrent patellar instability may occur in 15% to 40% of patients who have been treated nonoperatively for first-time patellar dislocations. Recurrent patellar poses a significant tremendous threat to patients’ well-being and a burden on the social economy. Various predisposing factors contribute to patellar instability, including femoral anteversion, external tibial torsion, genu valgum, trochlear dysplasia, patella alta, and vastus medialis obliquus atrophy.^4^

There are many ways to fix the graft on the patella in medial patellofemoral ligament (MPFL) reconstruction, including anchors, transpatellar tunnel fixation of tendons, and interference screw fixation.^5^, ^6^, ^7^, ^8^, ^9^ Fixation of ligament grafts with anchors is relatively simple and convenient, yet it is not very cost effective and carries the risk of anchor breakage and detachment.^5^ Transosseous tunnel fixation of the tendon is the most economical.^10^ However, both transosseous tunnel fixation and interference screw fixation of tendons can form larger medullary canals and bone defects, which increase the risk of patellar fractures.^11^^,^^12^ In this Technical Note, we merge the convenience of anchor technology with the economic advantages of transosseous tunnel fixation and introduce a technique of microbone tunnel technology (2-mm diameter) combined with high-strength sutures to fix the graft on the patella.

## Surgical Technique

Our technique is described in detail in Video 1 and in the following 5 steps.

### Step 1: Expose the Middle and Upper Third of the Medial Edge of the Patella

**Fig 1:**
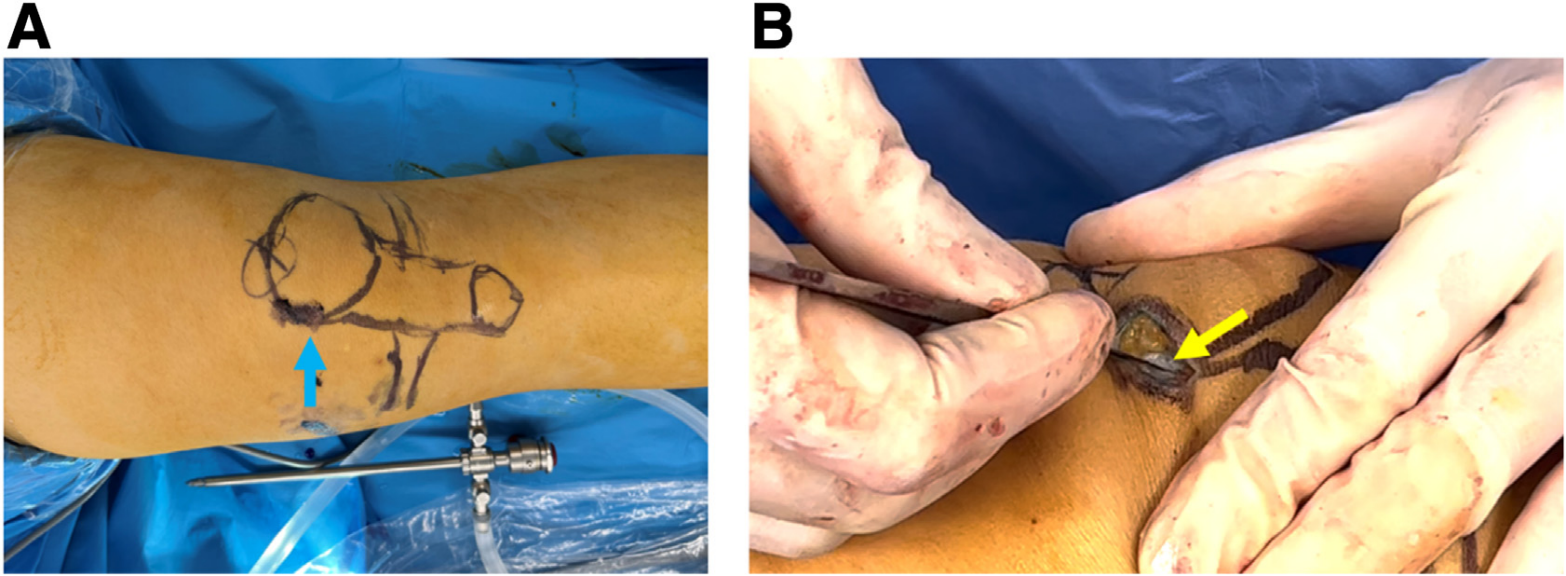
Surgical incision to expose patella of the right knee. (A) A longitudinal incision of about 2 cm is made centered on the middle and upper third of the medial patella. (B) Soft tissue is carefully separated layer by layer to expose the medial aspect of the patella. The patient is in a supine position with the right knee straight. Blue arrow: surgical incision markers; yellow arrow: deep fascia on the surface of the patella.

The patient is placed in the supine position for routine disinfection and draping. The autologous gracilis muscle graft is harvested, with the distal graft tendon stump secured using a 3-0 silk suture. The surgeon makes a 2-cm longitudinal incision in the middle and upper third of the medial edge of the patella (Fig 1A); cuts through the skin, subcutaneous tissue, and deep fascial tissue on the surface of the patella layer by layer to expose the bone surface of the patella; and then uses an arthroscopic shaver to remove excess subcutaneous fat tissue for better exposure the bony surface of the patella (Fig 1B).

### Step 2: Establish the Suture Fixation Structure for the Proximal Patella With a High-strength Suture

**Fig 2:**
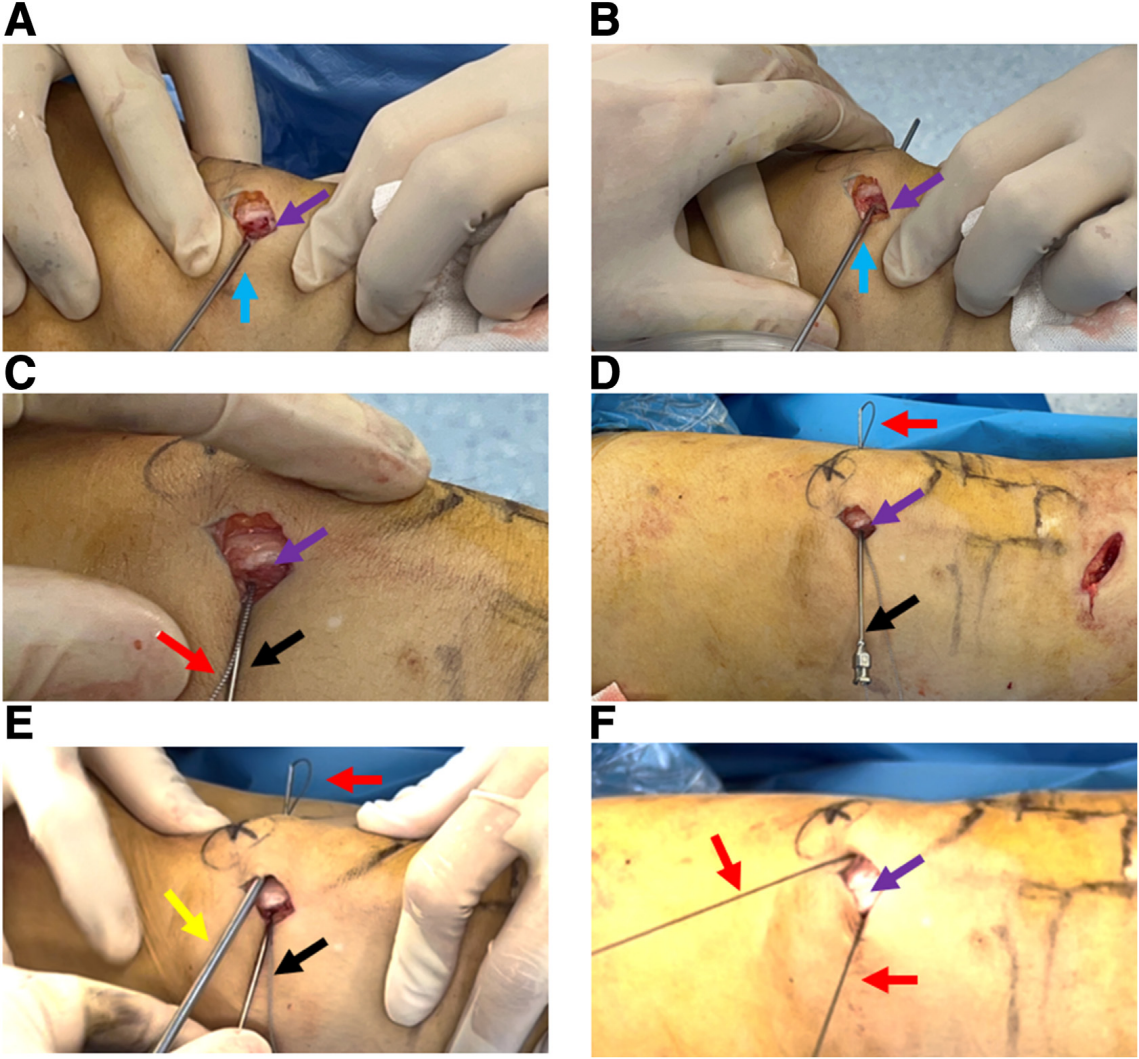
Preparation of a 2-mm microbone passage in the deep proximal patella and placement of high-strength sutures. (A) A 2-mm K-wire is positioned deep proximal in the upper third of the patella. (B) The K-wire is threaded diagonally from the deep proximal surface to the superficial lateral side of patella. (C) The spinal puncture needle is loaded with the high-strength suture. (D) Suture-loaded spinal puncture needle passes through the micro-osseous tract. (E) A grasper is used to grab one end of the high-strength suture subcutaneously. (F) The high-strength suture is pulled out subcutaneously. The patient is in a supine position with the right knee straight. Purple arrow: medial bony surface of the middle upper third of the patella; blue arrow: 2-mm K-wire; black arrow: spinal puncture needle; red arrow: high-strength suture; yellow arrow: the grasper.

**Fig 3:**
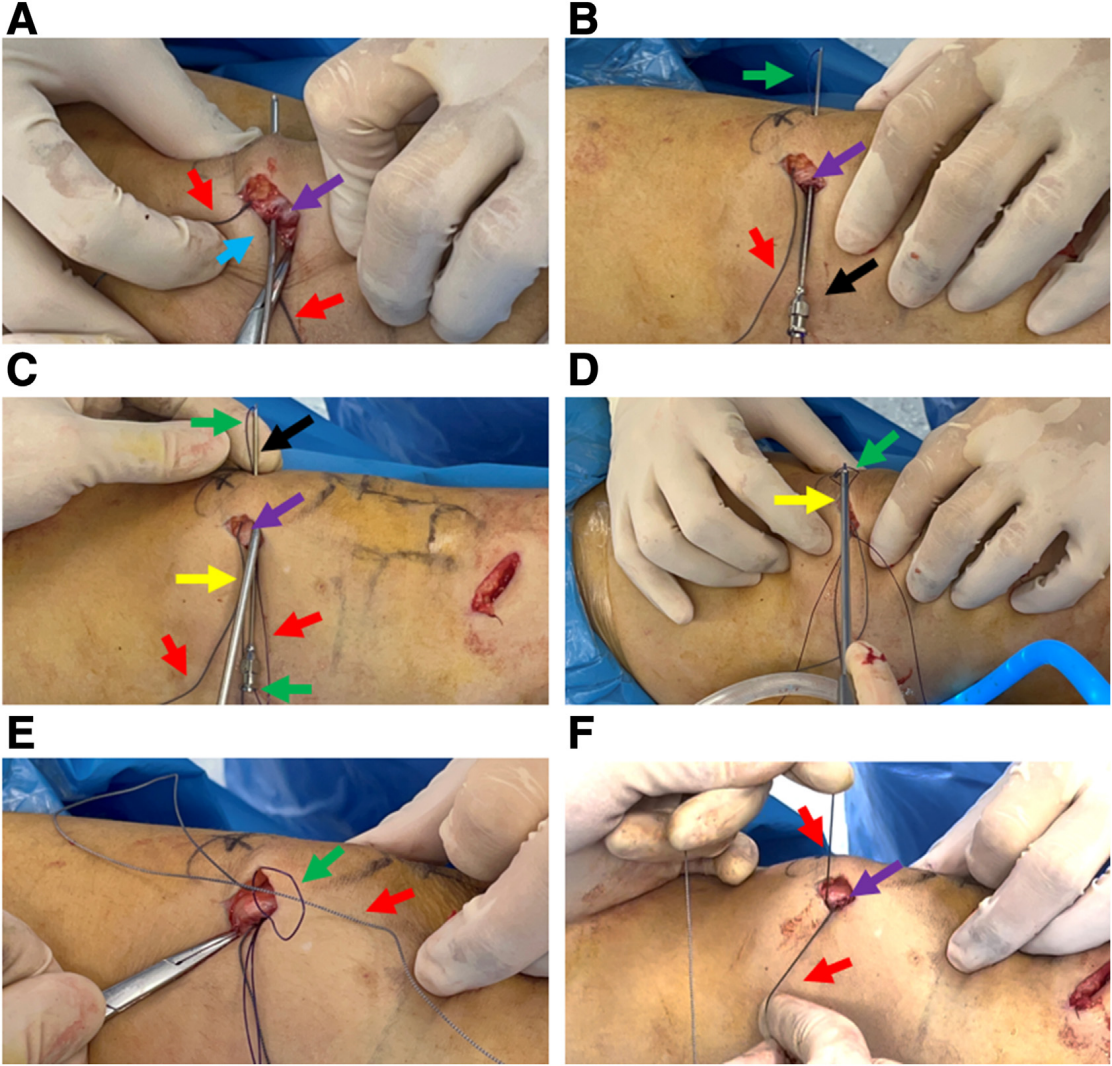
Preparation of proximal patellar graft fixation device. (A) A 2-mm K-wire is drilled in the proximal superficial aspect of the upper middle third of the patella and is threaded diagonally to the superficial lateral part. (B) The spinal puncture needle is loaded with PDS traction thread and passed subcutaneously along the micro-osseous tract to expose the loops on the lateral surface of the patella. (C) A grasper is used to grab the PDS loop percutaneously. (D) The grasper pulls out the PDS wire subcutaneously to the medial side of patella. (E) The PDS loop is attached to the subcutaneous end of the high-strength suture. (F) The PDS thread pulls the end of the high-strength suture from the proximal superficial bone tunnel and completes the preparation of the structure of the first high-strength suture device for graft fixation. Purple arrow: medial bony surface of the middle upper third of the patella. The patient is in a supine position with the right knee straight. Blue arrow: 2-mm K-wire; Black arrow: spinal puncture needle; Red arrow: High-strength line; Yellow arrow: the grasper; Green arrow: PDS thread. (PDS, polydioxanone.)

With an electric drill, a 2-mm Kirschner wire is obliquely drilled from the proximal and deep part of the exposed medial surface of the patella to the lateral surface of the patella to form a deep proximal bone tunnel (Fig 2 A and B, Table 1). The high-strength suture (ORTHOCORD Suture; No. 2 Violet W/ MO-7 1/2 Circle, Taper Point Needle, 22 mm; DePuy Mitek, Warsaw, IN) is loaded onto the spinal puncture needle and penetrates the skin from the medial surface of the patella along the microbone tract (Fig 2 C and D). During the procedure of spinal puncture needle-guided suture passing through the bone tunnel, caution should be taken to avoid suture cutting, which may impair the integrity of the suture, or even lead to the breakage of the suture (Table 1). The high-strength suture is retrieved subcutaneously from the lateral outlet of the bone canal by a grasper (Fig 2 E and F). The same method is used to prepare the bone tunnel at the superficial part of the proximal patella (Fig 3A). This time, a PDS line is loaded into the spinal puncture needle and threaded through the proximal superficial bone canal (Fig 3B). The suture grasper pulls a part of the PDS suture out of the lateral outlet of the bone canal to form a loop (Fig 3 C and D). The loop of the PDS line guides the high-strength suture at the lateral outlet of the proximal deep microbone tract (Fig 3E) and passes through the superficial bone canal to the medial side of patella to form a suture fixation structure for the proximal patella (Fig 3F).

**Table 1:** Pearls and Pitfalls of Transosseous Strengthened Fixation With High-strength Suture

Pearls	Pitfalls
The middle and upper third of the medial patella should be fully exposed.	The holes on the patella should not be too close; otherwise, there may be a risk of fusing into one hole.
The direction of the bone tunnel should be oblique to the superficial lateral part to avoid the K-wire being injected into the joint cavity when preparing the microbone tract.	When using spinal puncture needle to guide the suture through the bone tunnel, suture cutting should be avoided.
Guiding the suture by a spinal puncture needle is a simple and repeatable method.	
In order to accurately grasp the suture subcutaneously, skills are required when using the grasper. Surgeons can first grasp the body of the spinal puncture needle with a grasper and then withdraw the needle—the grasper would naturally hook the suture—and then pull the suture out subcutaneously.	
When the patella is exposed, the surface fascia should be protected to ensure that there is enough soft tissue to cover the patella when suturing and fixing.

### Step 3: Establish Suture Fixation Structure for the Distal Patella With a High-Strength Suture

**Fig 4:**
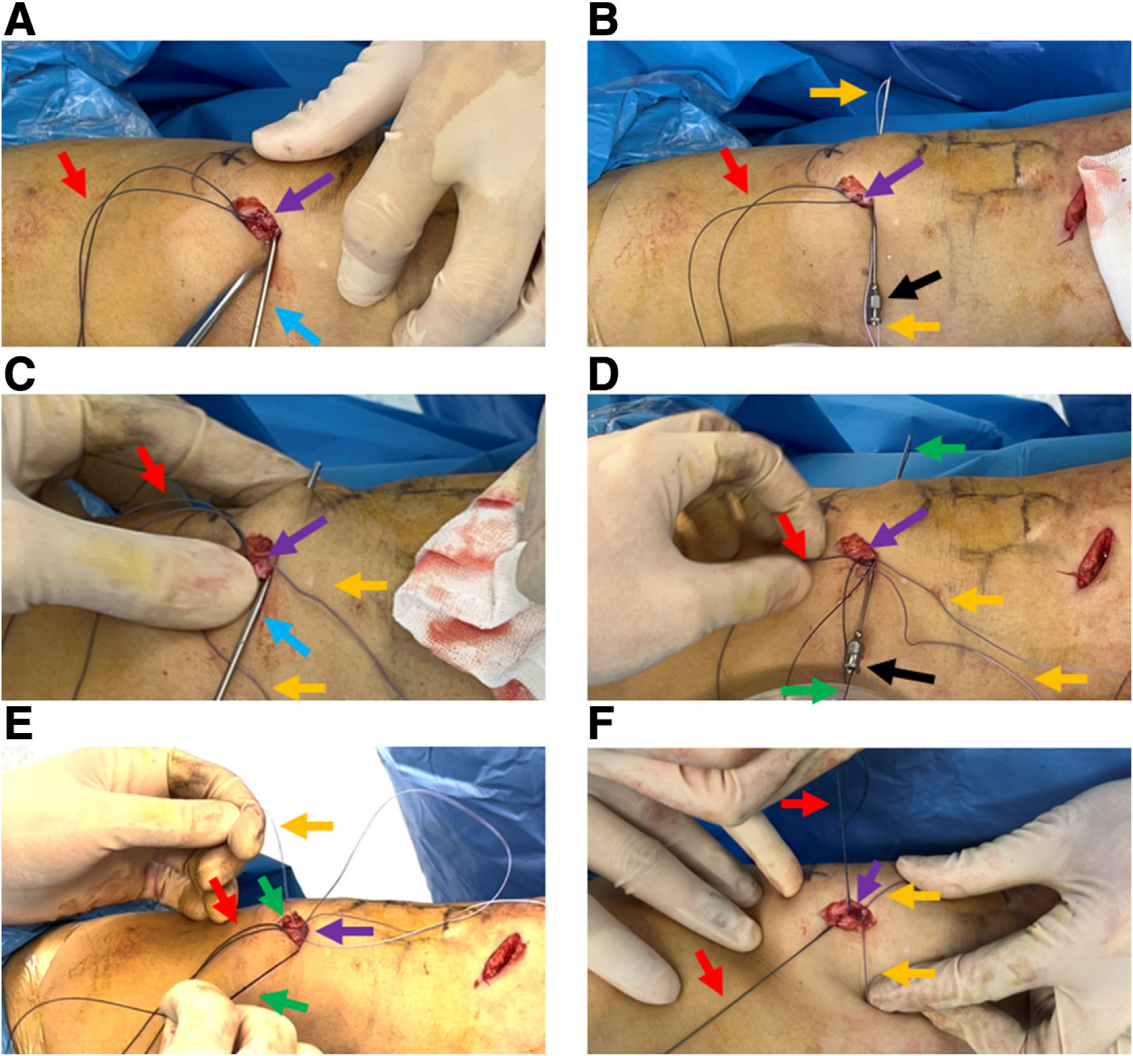
Preparation of 2-mm microbone tract at the distal end of the patella and placement of high-strength suture. (A) A 2-mm K-wire is drilled deep in the distal upper third of the patella, obliquely through the superficial lateral part. (B) The spinal puncture needle is loaded with a second high-strength suture, which is threaded along the microbone tract to expose the loop, and pulled out percutaneously with a grasper. (C) A 2-mm K-wire is drilled in the distal superficial part of the middle and upper third of the patella, and is threaded diagonally to the superficial lateral part. (D) The spinal puncture needle is loaded with a PDS thread and inserted along the superficial microbone tract to expose the loop. (E) The grasper pulls out the PDS loop percutaneously and uses the loop to guilde the tail end of the high-strength suture through the superficial microosseous tract. (F) The suture fixation structure with built-in high-strength suture in the patellar microbone tract is prepared. The patient is in a supine position with the right knee straight. Purple arrow: medial bony surface of the middle upper third of the patella; blue arrow: 2 mm K-wire; black arrow: spinal puncture needle; red arrow: proximal high-strength line; orange arrow: distal high-strength line; yellow arrow: Wire grasper. (PDS, polydioxanone.)

Next, a similar method is used to create 2 thin bone channels at the distal end of the patella near the middle of the patella. Another high-strength suture is passed percutaneously through the 2 thin bone canals (Fig 4 A and B), and the 2 ends are passed through the medial exits of the deep and superficial bone canals, respectively, with the PDS guiding loop to form a suture fixation structure for the distal end of the patella (Fig 4 C-F).

### Step 4: Fix the Autogenous Gracilis Tendon With the High-Strength Suture on the Proximal and Distal Patella

**Fig 5:**
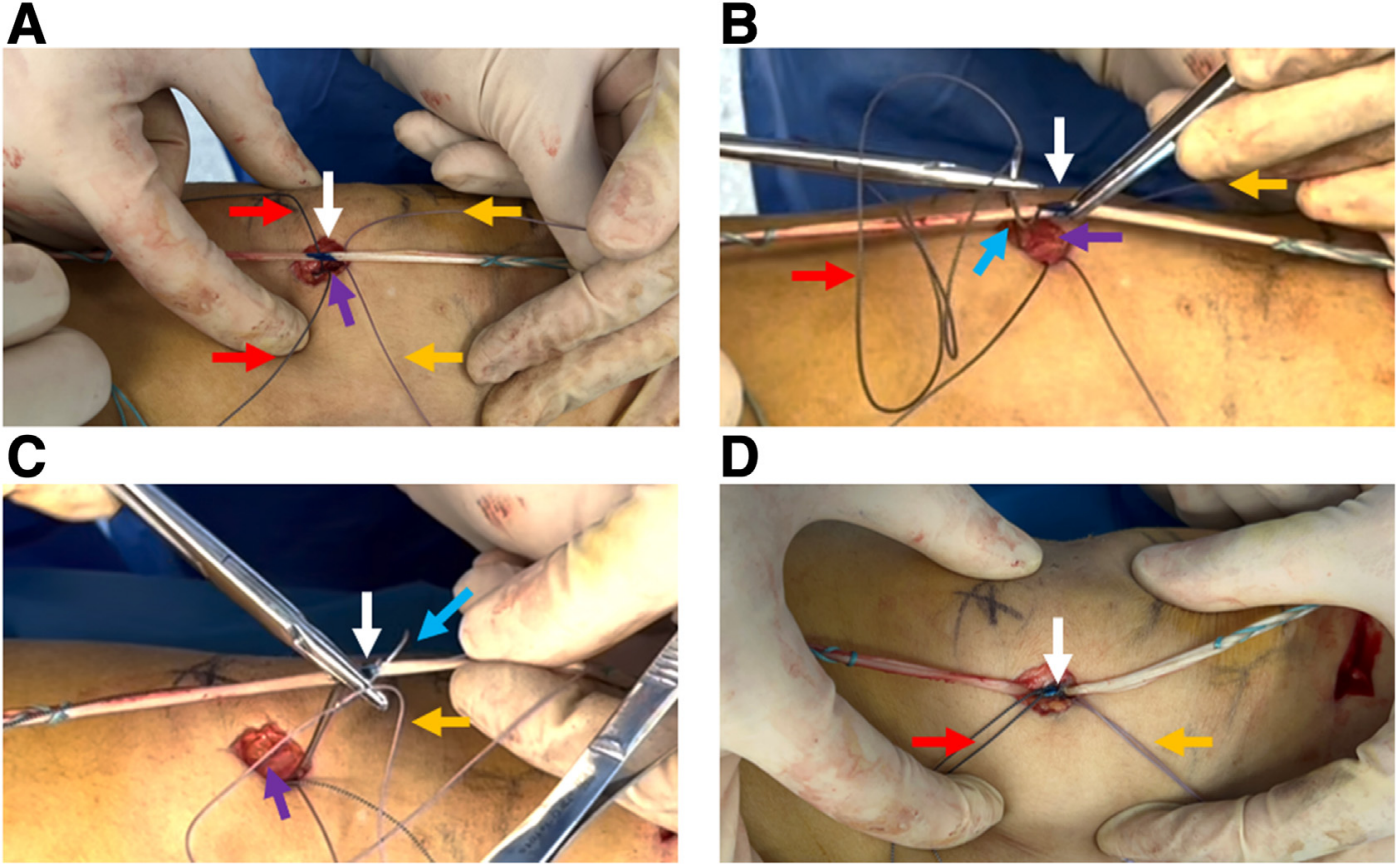
The fixation of the tendon graft using prepared built-in high-strength suture. (A) The gracilis tendon midpoint is marked and aligned with the center point of the 4 tunnel exits. Using the midpoint of the gracilis tendon as the marker, the tendon is braided with the ends of the high-strength suture on the proximal (B) and distal (C) parts, and each suture end is sutured 3 times on the tendon. (D) Two high-strength sutures are knotted to secure the gracilis tendon against the bony surface of the medial border of the patella. The patient is in a supine position with the knee straight. Purple arrow: medial bony surface of the middle upper third of the patella; white arrow: gracilis tendon graft; blue arrow: round needle; red arrow: proximal high-strength suture end; orange arrow: distal high-strength suture end.

The 2 ends of the autogenous gracilis tendon are conventionally braided, and the midpoint of the tendon is marked (Fig 5A). The midpoint of the gracilis tendon is aligned with the midpoint of the medial outlet of the proximal and distal patellar canals (Fig 5A). Each end of the high-strength suture at the medial outlets is continuously sutured to the prepared tendon 3 times (Fig 5 B and C). The sutures are tightened to ensure that the middle part of the tendon is attached to the surface of the patella. High-strength suture at the proximal and distal ends are then knotted separately to secure the tendon (Fig 5D).

### Step 5: Suture the Deep Fascia to Strengthen the Fixation of the Tendon Graft

**Fig 6:**
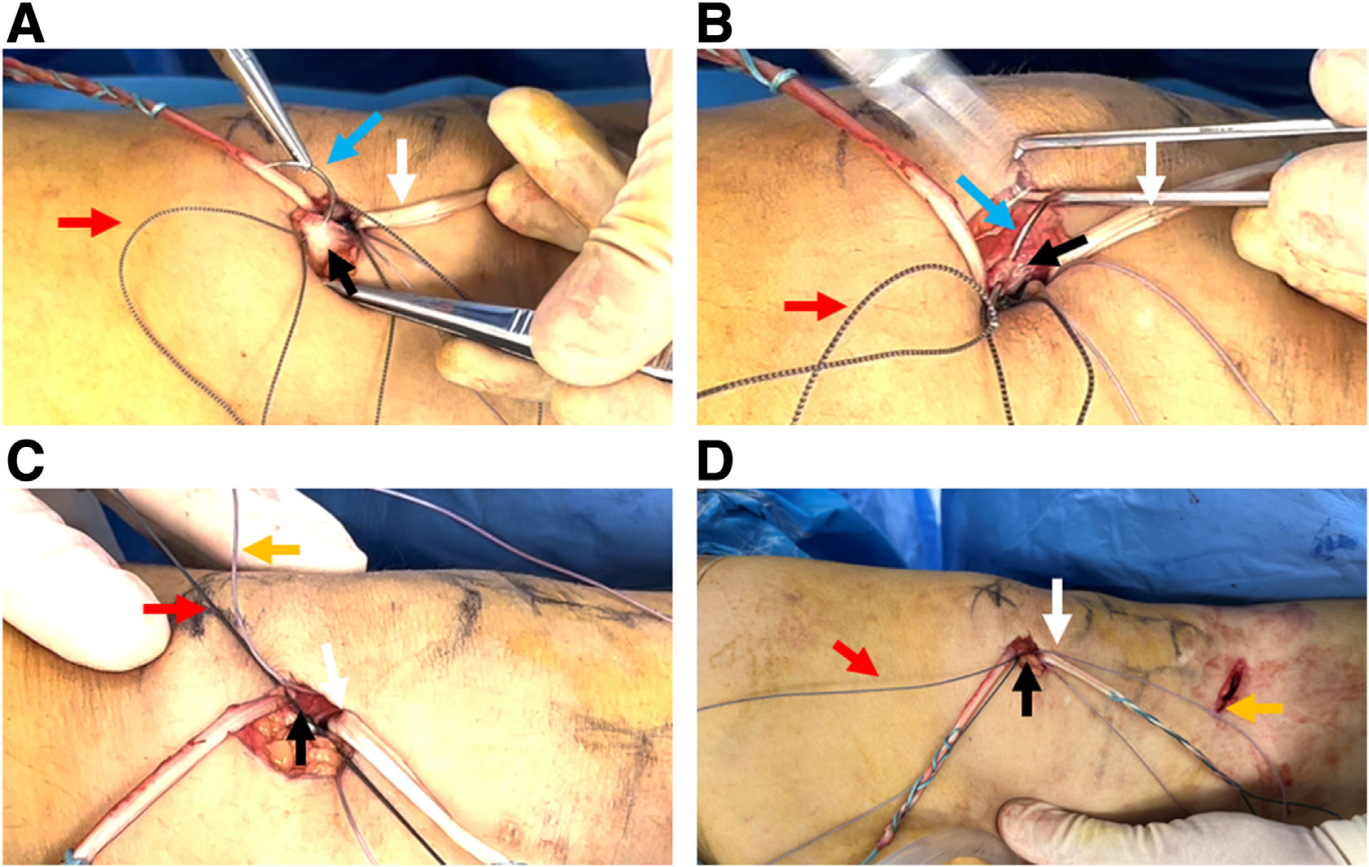
Deep fascial suture to strengthen the fixed tendon. (A) Deep fascia on the patellar surface is sutured with one caudal end of the proximal high-strength suture. (B) Deep fascia on the patellar surface is sutured with the other caudal end of the proximal high-strength suture. (C) The proximal high-strength suture ends are knotted. (D) The distal high-strength suture ends are sutured on the deep fascia to close the incision on the patellar surface to strengthen the fixation of the tendon graft. The patient is in a supine position with the right knee straight. White arrow: gracilis tendon graft; blue arrow: round needle; red arrow: proximal high-strength line; orange arrow: distal high-strength line; black arrow: deep fascia on the surface of the patella.

Finally, the 4 ends are sutured to the deep fascia on the surface of the patella to close the deep fascial incision (Fig 6 A and B). By suturing the fascial using the suture ends, the fixation of the tendon graft on the patella is strengthened (Fig 6 C and D).

## Discussion

MPFL repair and reconstruction are the primary surgical interventions for treating patellar dislocations.^13^^,^^14^ Acute patellar dislocation for the first time is usually treated conservatively, whereas MPFL repair is generally performed on patients with severe rupture at the attachment points of ligament.^14^ As for patients with recurrent patellar dislocation and chronic patellar dislocation with a long natural history, MPFL repair may not be suitable, evidenced by a high failure rate.^15^^,^^16^ MPFL reconstruction is an effective surgical procedure for the treatment of patellofemoral instability, which is safe and effective in patients of all ages, without marked predisposing anatomic abnormalities and moderate/severe osteochondral lesions.^17^

A meta-analysis that examined 21 studies found that approximately 43% of patients who underwent the double transpatellar tunnel fixation technique complained about consistent palpable pain at the medial patellar edge.^18^ In addition, although the transpatellar tunnel fixation increased patellar stability, the compression on the joint also increased.^19^ The transpatellar tunnel fixation also increased the stiffness of grafts, compared with suture anchor fixation, after increased tension and tunnel malpositioning.^20^^,^^21^ Our technique using 2 high-strength sutures to establish the fixation system through microbone tracts takes the advantage of anchor fixation by mimicking the natural attachment and biomechanics of MPFL (Table 2) to achieve reliable fixation.

**Table 2:** Advantages and Disadvantages of Transosseous Strengthened Fixation With High-strength Suture

Advantages	Disadvantages
Fixation materials are more economic.	The operation is more complex than anchor techniques.
Implants are less invasive.	The risk of suture cutting is inevitable when the suture is guided through the microbone tract by the spinal puncture needle.
Less damage to the patella.	
Minimally invasive incision for the exposure of patella.	
Strengthened fixation of tendon grafts by suturing the deep fascia using the suture ends.	
Less likely to cause technique-associated complications, such as patella fracture, joint disturbance by implants, and anchor pullout.	

Patellar fracture is a significant concern after MPFL reconstruction. Strategies for transverse patellar tunnels with looped grafts for patellar fixation, generally using 4.5-mm tunnels, have been associated with an increased risk for patellar fracture, caused by cortical violation of the transosseous tunnel of the patella.^22^ Deasey et al.^23^ reported a technique of small, short, oblique patellar tunnels for transverse patellar fixation of grafts, using 3.2-mm tunnels, and found that this technique and the suture anchor technique both resulted in a low incidence of patellar fracture, approximately 0.47% and 0%, respectively. The diameter of the bone tunnel in our study is only 2 mm, which causes less damage to the patella and avoids patellar fracture (Table 2). Our technique avoids invasive implants and reduces patella damage, which would theoretically decrease the risk of knee pain and inadvertent insertion of implants into joints^24^ (Table 2).

In addition, a systematic review summarized the mechanism of failure for suture anchor fixation and reported that the most common mechanism of failure was anchor pullout in suture anchor, especially with the use of titanium anchors.^25^ Greater pullout strength is generally associated with greater bone mineral density.^26^^,^^27^ Therefore, the application of suture anchors on older patients or patients with osteoporosis may have greater risk of anchors pullout, whereas our technique has no risk of implants pullout (Table 2). Moreover, after graft fixation using suture ends, the suture could be further applied to resuturing of the deep fascia on the surface of the patella, increasing the strength of graft fixation.

The disadvantage of our technique should also be mentioned. For example, the operation steps are relatively cumbersome compared with anchor placement. There is a risk of suture cutting when using a spinal puncture needle to guide the suture through the bone tunnel (Table 2). We would further conduct a cohort study with larger samples to investigate the efficiency and complications of our technique.

In conclusion, we introduce a technique for MPFL reconstruction on the patellar side designed to achieve biomechanical repair of the MPFL. Four microbone tracts with 2 high-strength sutures make up the graft fixation device to effectively rebuild the attachment of MPFL on the patella, with minimized damage to the patella. In addition, resuturing of the deep fascia on the surface of the patella with the suture ends further strengthens the graft fixation.

## Disclosures

All authors (Y.P., H.W., W. Yang, W. Yu, C.M., and W.H.) declare that they have no known competing financial interests or personal relationships that could have appeared to influence the work reported in this paper.

## Supplementary Data

Video 1 Surgical procedure of the microbone tracts technique for medial patellofemoral ligament reconstruction on the patella side with a high-strength suture. The video demonstrates the process of the medial patellofemoral ligament reconstruction on a right knee of the patient positioned supine with the knee straight.

## References

[1] Stefancin J.J., Parker R.D. (2007). First-time traumatic patellar dislocation: A systematic review. Clin Orthop Relat Res.

[2] Gravesen K.S., Kallemose T., Blønd L., Troelsen A., Barfod K.W. (2018). High incidence of acute and recurrent patellar dislocations: A retrospective nationwide epidemiological study involving 24.154 primary dislocations. Knee Surg Sports Traumatol Arthrosc.

[3] Hsiao M., Owens B.D., Burks R., Sturdivant R.X., Cameron K.L. (2010). Incidence of acute traumatic patellar dislocation among active-duty United States military service members. Am J Sports Med.

[4] Reagan J., Kullar R., Burks R. (2015). MPFL reconstruction: Technique and results. Orthop Clin North Am.

[5] Zampieri A., Girardin C., Hocquet B. (2023). Patellar dislocation recurrence after pediatric MPFL reconstruction: Bone tunnels and soft tissues versus suture anchors and interference screw. Orthop Traumatol Surg Res.

[6] Migliorini F., Driessen A., Quack V., Schenker H., Tingart M., Eschweiler J. (2020). Patellar fixation graft via suture anchors versus tunnel techniques during isolated MPFL reconstruction for recurrent patellofemoral instability: A systematic review of the literature. Arch Orthop Trauma Surg.

[7] Sasaki E., Kimura Y., Sasaki S., Yamamoto Y., Tsuda E., Ishibashi Y. (2022). Clinical outcomes of medial patellofemoral ligament reconstruction using FiberTape and knotless SwiveLock anchors. Knee.

[8] Raoulis V.A., Zibis A., Chiotelli M.D. (2021). Biomechanical evaluation of three patellar fixation techniques for MPFL reconstruction: Load to failure did not differ but interference screw stabilization was stiffer than suture anchor and suture-knot fixation. Knee Surg Sports Traumatol Arthrosc.

[9] Runer A., Klotz S., Schneider F. (2024). Medial patellofemoral ligament (MPFL) reconstruction using pedicled quadriceps tendon autograft yields similar clinical and patient-reported outcomes but less donor-site morbidity compared with gracilis tendon autograft. Arthroscopy.

[10] Siebold R., Chikale S., Sartory N., Hariri N., Feil S., Pässler H.H. (2010). Hamstring graft fixation in MPFL reconstruction at the patella using a transosseous suture technique. Knee Surg Sports Traumatol Arthrosc.

[11] Zhang H., Ye M., Liang Q. (2020). Clinical outcomes after medial patellofemoral ligament reconstruction with suture fixation of the gracilis tendon via transosseous tunnels. Orthop J Sports Med.

[12] Lenschow S., Schliemann B., Gestring J., Herbort M., Schulze M., Kösters C. (2013). Medial patellofemoral ligament reconstruction: Fixation strength of 5 different techniques for graft fixation at the patella. Arthroscopy.

[13] Nwachukwu B.U., So C., Schairer W.W., Green D.W., Dodwell E.R. (2016). Surgical versus conservative management of acute patellar dislocation in children and adolescents: A systematic review. Knee Surg Sports Traumatol Arthrosc.

[14] Ahmad C.S., Shubin Stein B.E., Matuz D., Henry J.H. (2000). Immediate surgical repair of the medial patellar stabilizers for acute patellar dislocation. A review of eight cases. Am J Sports Med.

[15] Camp C.L., Krych A.J., Dahm D.L., Levy B.A., Stuart M.J. (2010). Medial patellofemoral ligament repair for recurrent patellar dislocation. Am J Sports Med.

[16] Arendt E.A., Moeller A., Agel J. (2011). Clinical outcomes of medial patellofemoral ligament repair in recurrent (chronic) lateral patella dislocations. Knee Surg Sports Traumatol Arthrosc.

[17] Han H., Xia Y., Yun X., Wu M. (2011). Anatomical transverse patella double tunnel reconstruction of medial patellofemoral ligament with a hamstring tendon autograft for recurrent patellar dislocation. Arch Orthop Trauma Surg.

[18] Heo J.W., Ro K.H., Lee D.H. (2019). Patellar redislocation rates and clinical outcomes after medial patellofemoral ligament reconstruction: Suture anchor versus double transpatellar tunnel fixation. Am J Sports Med.

[19] Arendt E.A. (2009). MPFL reconstruction for PF instability. The soft (tissue) approach. Orthop Traumatol Surg Res.

[20] Elias J.J., Cosgarea A.J. (2006). Technical errors during medial patellofemoral ligament reconstruction could overload medial patellofemoral cartilage: A computational analysis. Am J Sports Med.

[21] Bollier M., Fulkerson J., Cosgarea A., Tanaka M. (2011). Technical failure of medial patellofemoral ligament reconstruction. Arthroscopy.

[22] Schiphouwer L., Rood A., Tigchelaar S., Koëter S. (2017). Complications of medial patellofemoral ligament reconstruction using two transverse patellar tunnels. Knee Surg Sports Traumatol Arthrosc.

[23] Deasey M.J., Moran T.E., Lesevic M., Burnett Z.R., Diduch D.R. (2020). Small, short, oblique patellar tunnels for patellar fixation do not increase fracture risk or complications in MPFL reconstruction: A retrospective cohort study. Orthop J Sports Med.

[24] Shamrock A.G., Day M.A., Duchman K.R., Glass N., Westermann R.W. (2019). Medial patellofemoral ligament reconstruction in skeletally immature patients: A systematic review and meta-analysis. Orthop J Sports Med.

[25] Onggo J.R., Babazadeh S., Pai V. (2022). Smaller gap formation with suture anchor fixation than traditional transpatellar sutures in patella and quadriceps tendon rupture: A systematic review. Arthroscopy.

[26] Pietschmann M.F., Fröhlich V., Ficklscherer A. (2009). Suture anchor fixation strength in osteopenic versus non-osteopenic bone for rotator cuff repair. Arch Orthop Trauma Surg.

[27] Tingart M.J., Apreleva M., Zurakowski D., Warner J.J.P. (2003). Pullout strength of suture anchors used in rotator cuff repair. J Bone Joint Surg Am.

